# Supplementary material for: Human adipose stromal cells differentiate towards a tendon phenotype with adapted visco-elastic properties in a 3D-culture system
Source: Biol Open. 2025 May 12;14(5):bio061911. doi: 10.1242/bio.061911 (PMC12091229; doi:10.1242/bio.061911)
Supplement: Supplementary information [file biolopen-14-061911-s1.pdf]

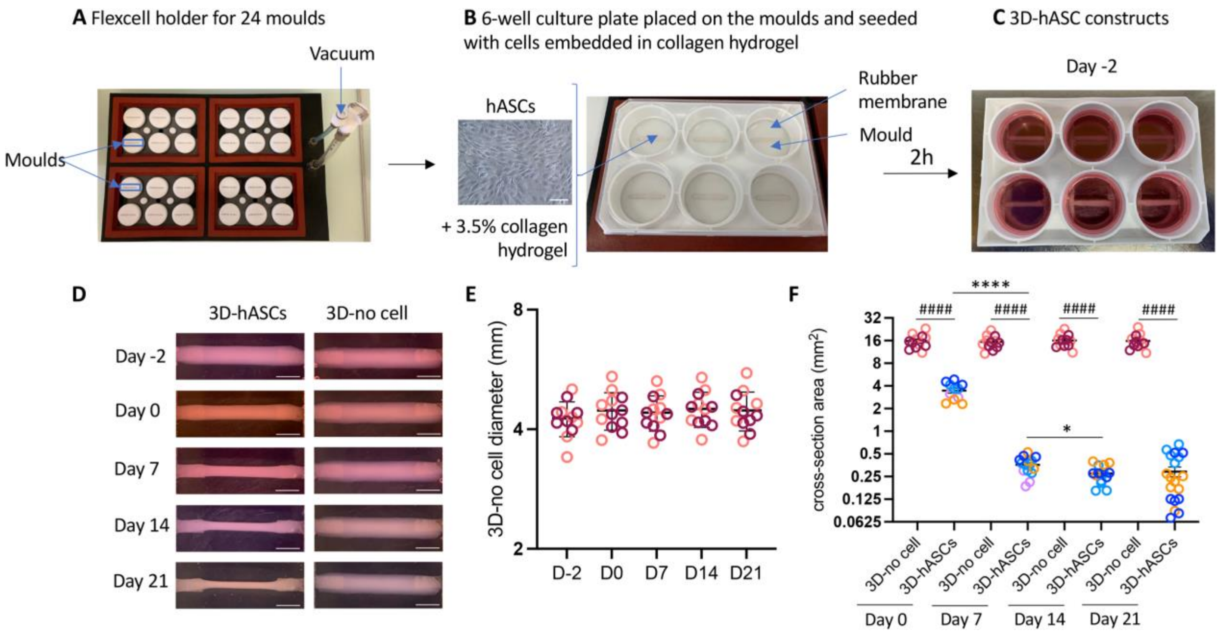


**Fig. S1. The Flexcell bioreactor.** A) This technology consists of a holder with 24 perforated cylindrical moulds connected to a vacuum pump and controlled by computer. B) Four 6-well plates can be placed on these moulds. The bottom of the wells consists of a rubber membrane with two thick anchor points. When the vacuum is applied, the bottom of the wells takes the cylindrical shape of the moulds. hASCs embedded in 3.5% collagen hydrogel are seeded between the two anchor points and incubated at 37°C, 5% $CO_2$ for 2 hours. C) At the end of the 2 hours incubation, the vacuum is broken and the plate is removed from the moulds. The plate is left in the incubator at 37°C, 5% $CO_2$ for 48 hours before experimentation (Day -2 timepoint). D) Pictures of 3D-hASC constructs and 3D-no cell constructs not seeded with cells. Scale bars, 0.5 cm. E) Diameters were measured from constructs at Day 0 (n=12), Day 7 (n=12), Day 14 (n=12) and Day 21 (n=12). Each colour represents a set of experiments. 2 independent experiments were performed with n=6 biological replicates for each experiment. F) Cross-section areas of 3D-no cell and 3D-hASC constructs were calculated from diameters measurements presented Figure 1B: Day 0 (n=11), Day 7 (n=15), Day 14 (n=14) and Day 21 (n=19). Each colour represents a set of experiments. 4 independent experiments were performed with 3<n<8 biological replicates for each experiment. For 3D-hASC constructs, the p-values were obtained using the Mann-Whitney test compared to each following stage. * P<0.05, **** P<0.0001. $^{\#}$ indicates the p-values of cross-section areas of 3D-no cell versus 3D-hASC constructs, $^{\#\#\#\#}$ P <0.0001.

Biology Open • Supplementary information

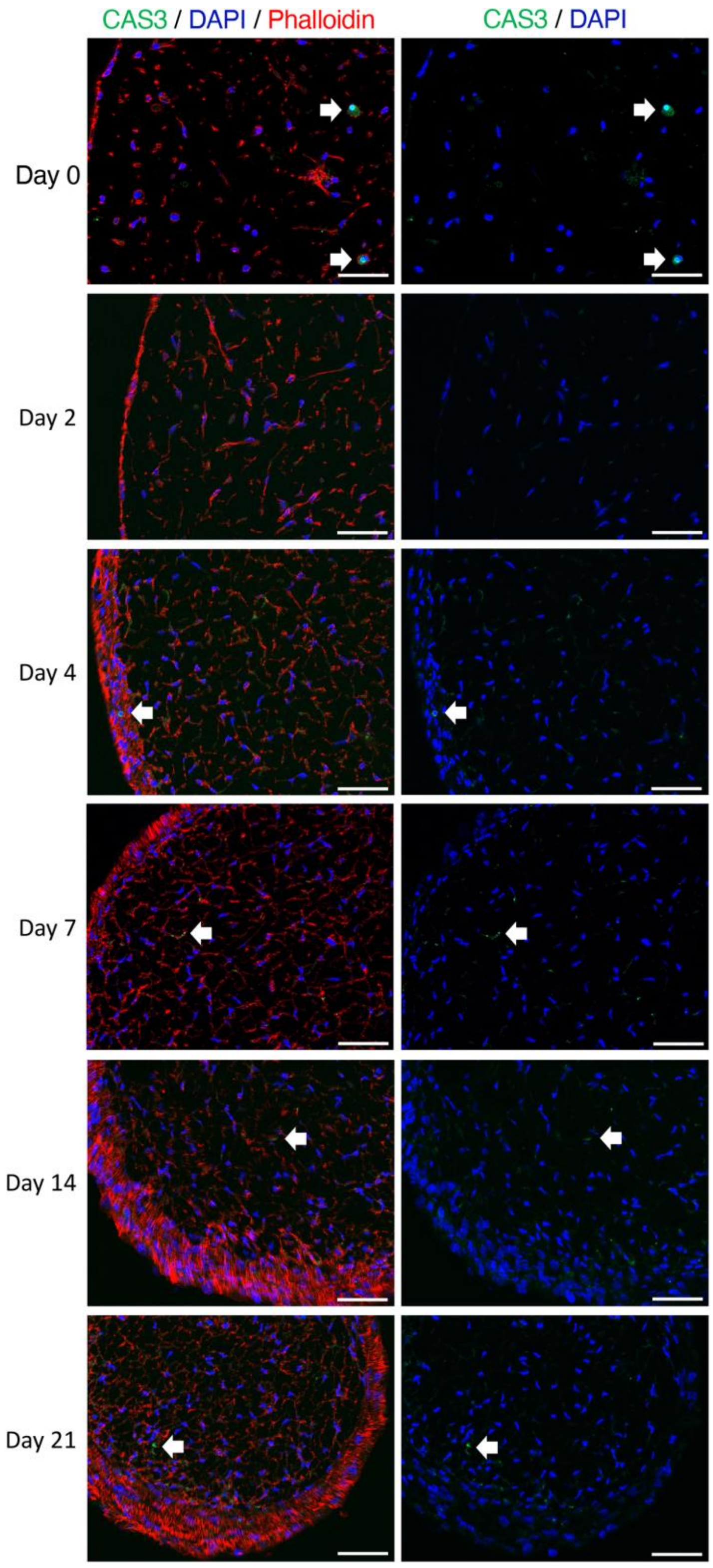


**Fig. S2. CASPASE labelling in 3D-hASC constructs over time.** A) 12µm transverse sections of 3D-hASC constructs on Day 0 and Day 21 of culture were stained with CAS3 antibody to detect apoptotic cells and DAPI/Phalloidin to visualise cell nuclei and cytoskeletal organisation. Scale bars 50 µm.

Biology Open • Supplementary information

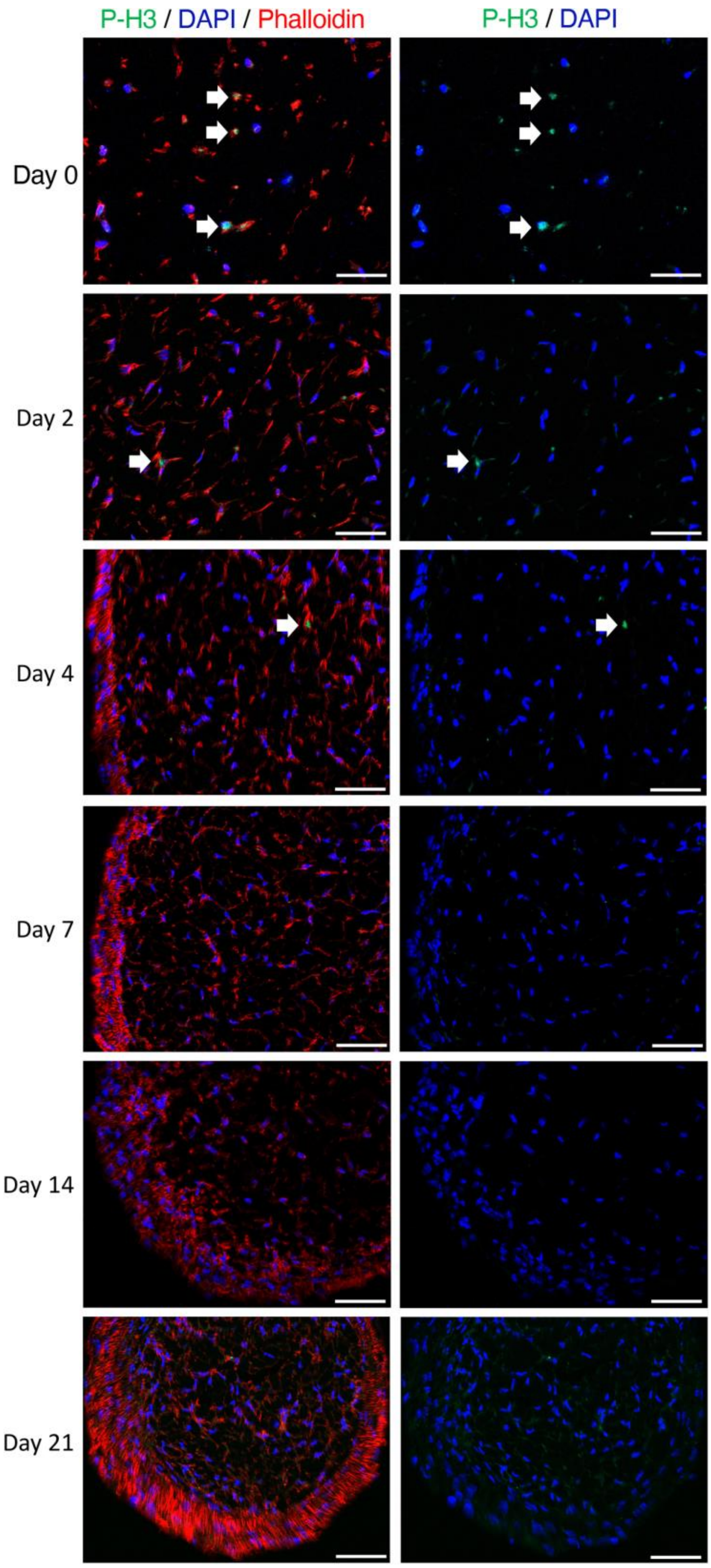


**Fig. S3. Phospho-Histone H3 labelling in 3D-hASC constructs over time.** A) 12μm transverse sections of 3D-hASC constructs on Day 0 and Day 21 of culture were stained with Phospho-Histone H3 antibody to detect proliferative cells and DAPI/ Phalloidin to visualise cell nuclei and cytoskeletal organisation. Scale bars 50 μm.

Biology Open • Supplementary information

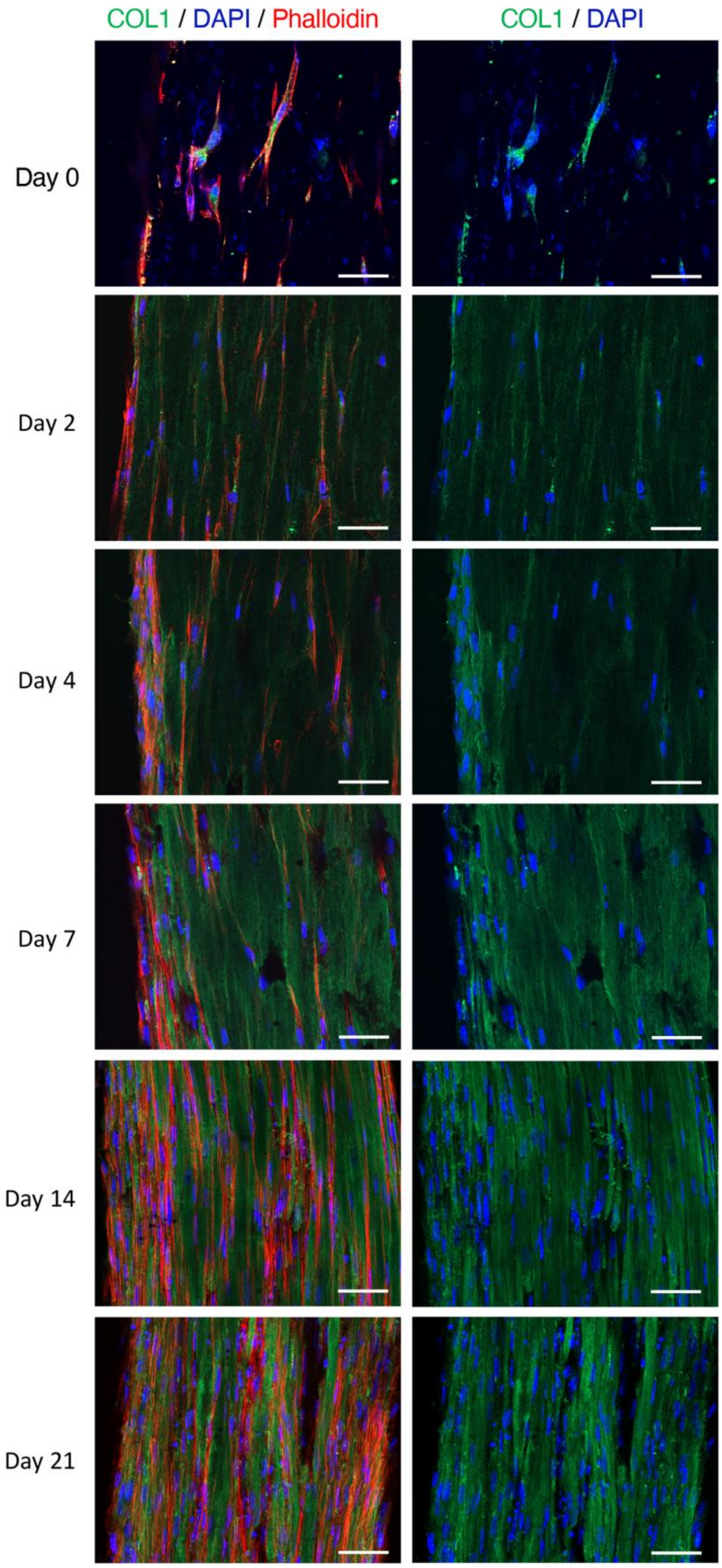


**Fig. S4. COL1A1 organisation in 3D-hASCs.** 12µm longitudinal sections of tendon constructs were performed on Day 0 and Day 2 and stained with DAPI/Phalloidin to visualise cell shape and COL1A1 antibody.

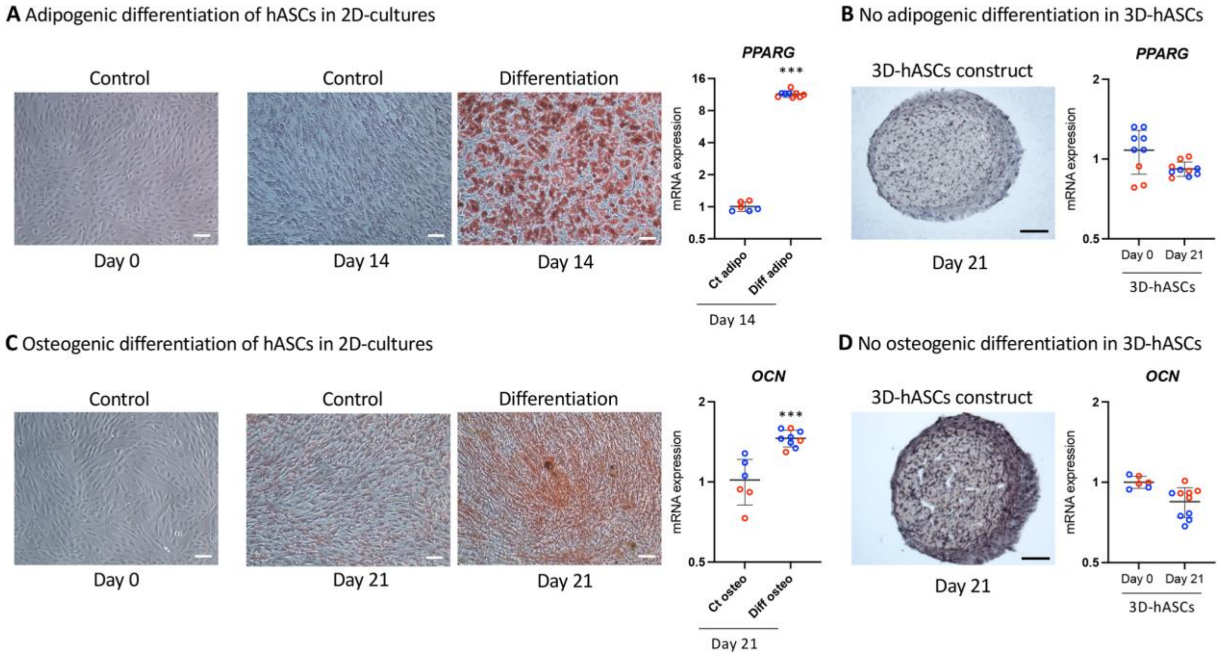


**Fig. S5. Adipogenic and Osteogenic differentiation of hASCs.** hASCs were subjected to A) Adipogenic differentiation for 13 Days. Control (cultured in basal medium) and differentiated hASCs were stained with Oil Red O and *PPARG* mRNA expression level was analysed by RT-qPCR. Scale bars 100µm. B) 21-Day 3D-hASC constructs were stained with Oil Red O and *PPARG* mRNA expression level was analysed by RT-qPCR. Scale bars 20µm. hASCs were subjected to C) Osteogenic differentiation for 14 Days. Control (cultured in basal medium) and differentiated hASCs were stained with Alizarin Red S and *OCN* mRNA expression level was analysed by RT-qPCR. Scale bars 100µm D) 21-Day 3D-hASC constructs were stained with Alizarin Red S and *OCN* mRNA expression level was analysed by RT-qPCR. Each differentiation experiment was performed 2 independent times with n ≥ 3 biological replicates. Scale bars 20µm.

Biology Open • Supplementary information

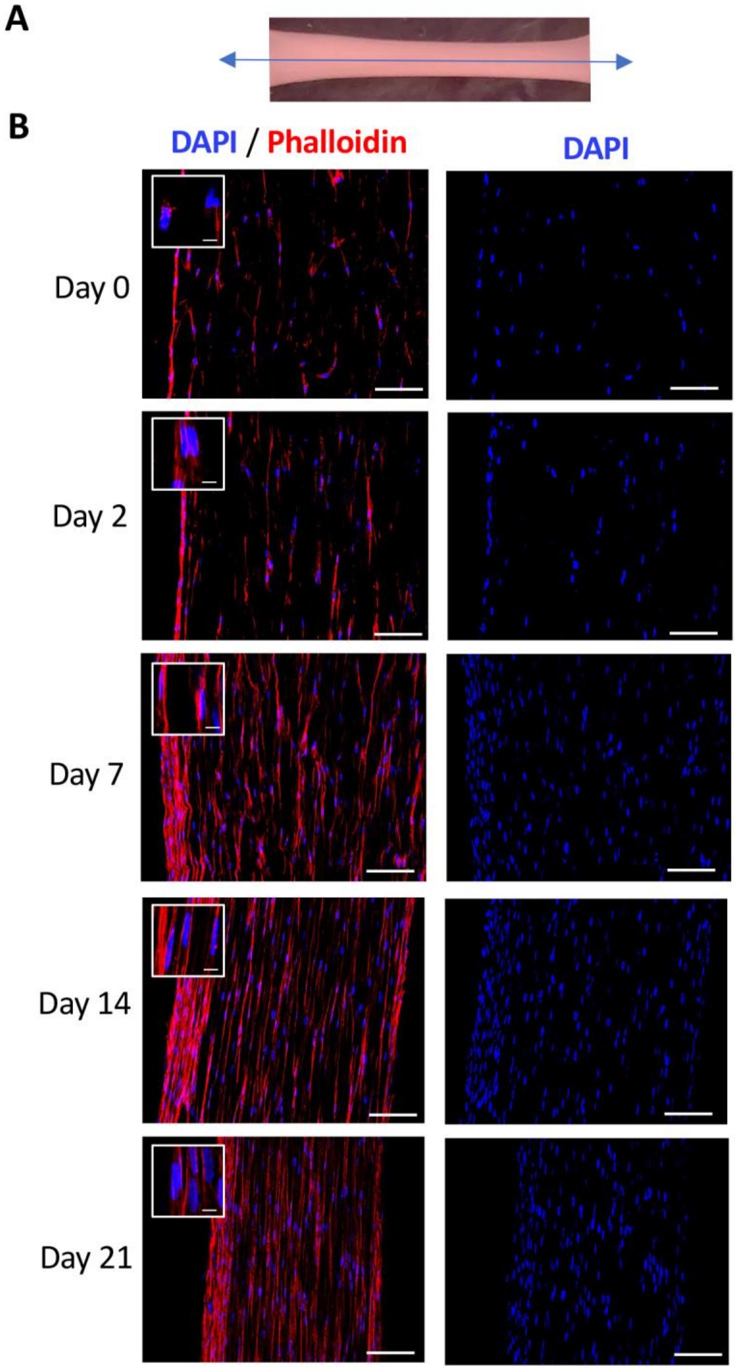


**Fig. S6. *COL3A1* mRNA expression in 3D-hASC constructs.** A) 3D-hASC constructs at Day 7 were transversally cryo-sectioned. 12 µm sections were hybridised with the DIG-labelled antisense probes for *COL3A1*. Scale bar: 500µm. B) 3D-hASC constructs at Day 0, Day 7, Day 14 and Day 21 were transversally cryo-sectioned. 12 µm sections were hybridised with the DIG-labelled antisense probes for *COL3A1*. Scale bars: 50µm.

Biology Open • Supplementary information

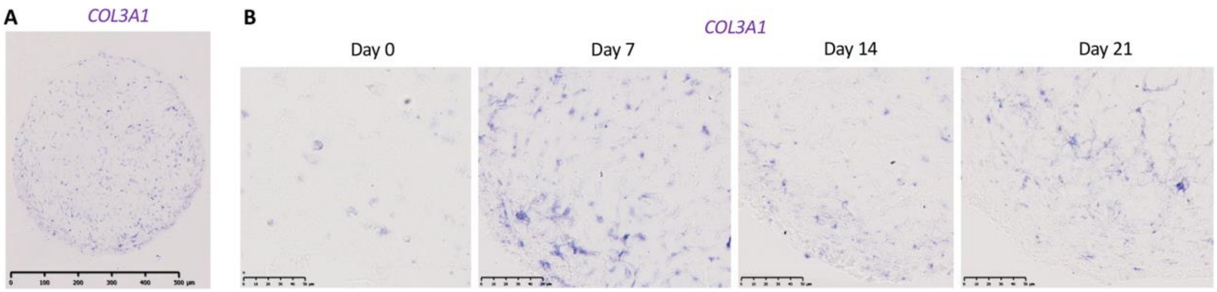


**Fig. S7. Cellular and nuclear organisation in 3D engineered 3D-hASCs.** A) 3D-hASCs longitudinal section representation. B) Longitudinal sections of 3D-hASC constructs were performed on Day 0, Day 2, Day 4, Day 7, Day 14 and Day 21 3D-constructs and stained with DAPI/Phalloidin to visualise cell shape. Scale bars: 200 µm. White squares represent higher magnification. Scale bars: 100µm.

Biology Open • Supplementary information

**Table S1.**

| | RT-qPCR Forward primer | RT-qPCR Reverse primer | Reference |
|---|---|---|---|
| ***hANXA1*** | AGTTCTTTGCAAGAAGGTAGAGA | CTGATCCGGGACCACCTTTG | Designed in the lab |
| ***hCOL1A1*** | GATGGCTGCACGAGTCACAC | GTATTCAATCACTGTCTTGCCCC | Designed in the lab |
| ***hCOL2A1*** | TGGCTGACCTGACCTGATGTCC | TGCAGTCTGCCCAGTTCAGGTC | Designed in the lab |
| ***hCOL6A3*** | AACATCGGCACTTGCCCTTA | ATATCAGCAGCCGCACCATT | Designed in the lab |
| ***hCOL14A1*** | ACTCCGAGGGAAGAGAGCAA | TACATGGGGTGTAGCAGCCA | Designed in the lab |
| ***hDPT*** | TGTCGCTACAGCAAGAGGTG | GTGGTTGTTGCTCCTCGGAT | Designed in the lab |
| ***hHIC1*** | TCCCCAGTCCCAGAAACAGA | CTTTCTGTCCCGTTTGCAGC | Designed in the lab |
| ***hMKX*** | CATCGTCATCAGAACTGAAGGCA | TCTGTTAGCTGCGCTTTCACCC | (Bayer *et al.*, 2014) |
| ***hOCN*** | GGCGCTACCTGTATCAATGG | TCAGCCAACTCGTCACAGTC | Designed in the lab |
| ***hPDGFRA*** | TGTGGGACATTCATTGCGGA | AAGCTGGCAGAGGATTAGGC | Designed in the lab |
| ***hPOSTN*** | GAGGAAGTTGCAAGCCAACA | CACTGAGAACGACCTTCCCT | Designed in the lab |
| ***hPPARG*** | AAGCCCTTCACTACTGTTGACT | CAGGCTCCACTTTGATTG | (Waldner *et al.*, 2018) |
| ***hPRRX1*** | GACCATGACCTCCAGCTACG | TAGCAGGTGACTGACGGAGA | Designed in the lab |
| ***hS100A10*** | GGCTACTTAACAAAGGAGGACC | GAGGCCCGCAATTAGGGAAA | Głowacka et al., 2021 |
| ***hSCX*** | CCCAAACAGATCTGCACCTT | CGGTCCTTGCTCAACTTTCT | Designed in the lab |
| ***hTHBS2*** | GACACGCTGGATCTCACCTAC | GAAGCTGTCTATGAGGTCGCA | (Xu *et al.*, 2020) |
| ***hTM4SF1*** | CAGCCCTTGGCTTAGCAGAA | ACTCGGACCATGTGGAGGTA | Designed in the lab |
| ***hTNMD*** | AGCACTTCTGGCCGGAGG | AAGTGTGCTCCATGTCATAGGCT | Designed in the lab |
| ***hYWHAZ*** | CCGCTGGTGATGACAAGAAAGGGAT | AGGGCCAGACCCAGTCTGATAGGA | (Ragni *et al.*, 2013) |

Biology Open • Supplementary information

| Gene name | In situ hybridisation probe Forward primer | In situ hybridisation probe T7-Reverse primer | Reference |
|---|---|---|---|
| ***hCOL1A1*** | CTCCCCAGCTGTCTTATGGC | TAATACGACTCACTATAGGGCGCACCATCATTTCCACGAGC | Designed in the lab |
| ***hCOL3A1*** | CCTACTCGCCCTCCTAATGG | TAATACGACTCACTATAGGGCTCGAAGCCTCTGTGTCCTTT | Designed in the lab |
| ***hSCX*** | GGTCGCTACCTGTACCCTGA | TAATACGACTCACTATAGGGCCTGAGGCAGAAGGTGCAGAT | Designed in the lab |
| ***hTHBS2*** | ACCAGGACAAAGACACGACC | TAATACGACTCACTATAGGGCCCACGTACATCCGGCTCTTT | Designed in the lab |
| ***hTNMD*** | TGGAAATGGCACTGATGAAA | TAATACGACTCACTATAGGGCCAGCATTGGGTCAAATTCAA | Designed in the lab |
